# Do It, Don’t Feel It, and Be Invincible: A Prolog of Exercise Addiction in Endurance Sports

**DOI:** 10.3389/fpsyg.2019.02692

**Published:** 2019-12-20

**Authors:** Abel Nogueira, Maribel Tovar-Gálvez, Juan González-Hernández

**Affiliations:** ^1^Department of Physical Education and Sports, Faculty of Physical Activity and Sport Sciences, University of León, León, Spain; ^2^Department of Nursing, Faculty of Health Sciences, University of Granada, Granada, Spain; ^3^Department of Personality, Evaluation and Psychological Treatment, Faculty of Psychology, University of Granada, Granada, Spain

**Keywords:** narcissism, Machiavellianism, psychopathy, grit, exercise addiction

## Abstract

The social relevance of endurance sports has increased people’s motivation to engage in these particular physical activities, associating their practice with a particular lifestyle (e.g., feeling victorious and a feeling of self-improvement). Therefore, the dark personality traits (not because they are negative but because they are more hidden), understood as a personal and adaptive response to the psychosocial relationships that athletes establish while practicing these sports. Following these arguments, Grit has been used to trace the response of athletes in their quest to improve performance and endurance in the face of common setbacks suffered as a result of long hours of training. Empirical studies should help to discover how these personality traits can pose real challenges to their adaptation, and what the impact of their psychological response may be in a functional or dysfunctional way [e.g., exercise addiction (EA)], in order to classify them as risk or protective factors. Through transversal design, the present study sought to explore the relationship between Grit and Dark Traits of Personality regarding the appearance of EA in a sample (*N* = 241) of amateur endurance sport athletes (*M*_age_ = 31.80; SD = 9.87). The results show that men not only score higher for addiction levels but also for narcissism (grandiosity feelings) and psychopathy (coldness) factors. If signs of narcissism and Machiavellianism increase, perseverance efforts grow too, and the likelihood of EA increases considerably. The conclusions drawn on the basis of the results allow us to place consistency of interest as a protective factor for the EA, whereas Dark Traits of personality – especially Machiavellianism – constitute a risk factor.

## Introduction

“The more the merrier” – it seems like a really common statement among amateur athletes, especially between those who practice endurance sports (e.g., 5 and 10-km runs, half-marathons, marathons, triathlons, ultra-endurance races, mountain trails, cycling, ironman races, etc.) and other recent sport modalities like CrossFit, which combines aerobic and anaerobic exercises with the ultimate goal of improving fitness and physical performance (Glassman, 2007; Belger, 2012; Lichtenstein and Jensen, 2016). The principal precursors for both modalities have been referenced by González-Hernández et al. (2019b), and are aligned with “trying to give the best of one-self combined with high intensity” (Simpson et al., 2017) and the achievement of new challenges in the search for unlimited improvement (Cruz, 2013).

In addition to these benefits, one of the greatest attractions of endurance sports is the speed with which these gains can be achieved (Lee et al., 2014) and the subjective perception of improvement that is entailed within. The practice of an endurance sport (e.g., running or cycling) implies a series of vigorous physical efforts, in which it is necessary to preserve resources and beliefs, maintained at high levels of energy and activation (Lukács et al., 2019). This is combined with the emergence of impulses and the consequent need to regulate them both, emotionally and behaviorally (González-Hernández et al., 2019a).

These sudden results (“*here and now*”) are maybe one of the main reasons why these sports have received a boost in popularity during the last decade (Knechtle et al., 2014; Lara et al., 2014). We live in a system that hardly enables us enough time to meet our fitness goals, and we try to compensate by signing into shorter but more intense training programs with the rationale of achieving visible benefits in the short/mid-term. On this matter, endurance sports (especially running or CrossFit) fit perfectly within today’s packed schedules because they provide high intensity training (Holt et al., 2014; Predel, 2014; Davies et al., 2016; Köteles et al., 2016; Lichtenstein and Jensen, 2016).

However, this can become a double-edged sword as the immediate benefits may lead toward creating unhealthy sports behaviors, such as “Exercise Addiction” (EA) (Nogueira et al., 2018). This concept is used to refer to any physical activity that is carried out excessively (an imbalance in the dose–response among exercise and health) without control and that has become the central axis in people’s lives (Landolfi, 2013; Kovacsik et al., 2018). As with the rest of the addictions, EA can be identified by six components (or patterns): *salience*, *mood modification*, *tolerance*, *withdrawal symptoms*, *personal conflict*, and *relapse* – i.e., the tendency to return to excessive activity after periods of abstinence or control (Brown, 1993; Griffiths, 2005).

The prevalence values, according to previous research, can alter depending on the instrument or the sample used to register the data [e.g., Exercise Dependence Scale-Revised (EDS-R), Symons Downs et al., 2004; or Exercise Addiction Inventory (EAI), Terry et al., 2004; Exercise Addiction Inventory-Revised (EAI-R), Szabo et al., 2019]. For this reason, and considering the literature surrounding this topic, we decided to go by EAI; the most recent studies show how this instrument seems like a more appropriate tool to screen the risk for EA in specific populations of sportspeople due to its capacity to identify a higher proportion of people at risk of EA (Di Lodovico et al., 2019).

In the present study, based on the EAI cut-off score for individuals considered at risk of EA (Terry et al., 2004; Sicilia-Camacho et al., 2013), 9.5% of the amateur athletes were classified to be at risk of EA (total EAI score 24–30), while only 3.3% were considered asymptomatic (total EAI score 0–12). The major part of the participants of this study were considered as symptomatic (total EAI score 13–23), with a percentage of 87.2%.

Among all studies into EA, there is a line of research that is focused on the study of personality traits that increase the predisposition of addictive behaviors toward physical exercise linked with characteristics like big five general factors (Andreassen et al., 2013), perfectionism (Bircher et al., 2017), narcissism (Cook et al., 2018), or neuroticism (Lichtenstein et al., 2017). These studies confirmed the direct and combined role of the personality traits as predictive or mediator factors in the development of EA (Bruno et al., 2014). In this sense, high levels of narcissism, perfectionism, neuroticism, and low self-esteem are related to risk of EA (Lichtenstein et al., 2017). The correlations among neuroticism, extroversion and conscientiousness (positive), and agreeableness and openness to experience (negative), however, were identified as indicators of this type of dysfunctional behavior (Andreassen et al., 2013).

Grit (González-Hernández et al., 2019b) and Dark Triad of Personality (DTP; González et al., 2018) are two of the most recent personality traits that have been incorporated into the analysis of endurance training for these sport disciplines.

There are two concepts that create the basis for Grit: *Passion*, understood as the result of hours and hours of practice, and *Perseverance*, the ability to withstand and overcome a large number of obstacles (Mills, 2017; Dumas and Perry-Smith, 2018). These are the main factors that motivate individuals to achieve long-term goals beyond talent, which ultimately defines what makes great winners special (Duckworth, 2016, p. 8; Duckworth et al., 2007). Grit has been analyzed in detail in other areas such as educational contexts (Duckworth et al., 2011) and military contexts (USMA, West Point) (Duckworth et al., 2007; Maddi et al., 2012; Kelly et al., 2014) before even being studied in the domain of sports. In sports, the two factors presently under discussion – perseverance and passion – are usually the attributes required to obtain the best possible performance (Gilchrist et al., 2017).

The DTP (Paulhus and Williams, 2002) groups together *Narcissism* (defined by feelings of vanity, superiority, dominance, dependent needs, or prominence), *Machiavellianism* (defined by the use of charm, manipulation, strategy, or misrepresentation toward others), and *Psychopathy* (defined by impulsiveness, emotional coldness, aggressiveness, or empathic withdrawal). Initially described as process features with negative connotations, in the sporting context, these have been linked to the search for improvement, according to the kind of response (functionally–dysfunctionally), resulting from a competitive vision that the athlete puts into practice at any time and in any circumstance based on his sporting experiences (Luciano Soriano et al., 2002; Houston et al., 2015).

As Starcevic and Khazaal (2017) point out, everything related to behavioral addictions induces some controversy because this type of addiction falls somewhere that is halfway between impulse-control disorders and substance abuse disorders.

According to this, it is necessary to develop studies that provide new points of view about these addictions. Considering all of this, the present study seeks to understand in what way what it is that an initially desired activity (exercise) can become a risk factor (addiction) for those who practice it, and how the personality traits (Dark Traits and Grit) can modify and influence the athlete–sport relationship (particularly for CrossFit and endurance sports) to the point of functionally/dysfunctionally affecting the psychological response. An increase in the indicators of weekly training and high levels of Dark Personality traits are shown as risk factors, while high levels of consistency in interest are protective of EA.

## Methods

### Procedure and Participants

Based on a cross-sectional, non-random, and descriptive design, it was requested through social networks that those who practice endurance sports (including recommending it to others) could respond to an *ad hoc* online survey through the website onlineencuestas.com. The structure of the questionnaires consists of two sections: the first section is used to collect socio-demographic information (e.g., gender, age, type of sport practiced, and amount of exercise per week), and the second section includes the three main scales of the study [the Short Grit Scale (Grit-S), the Short Dark Triad Scale (SD3), and the EAI] in their corresponding Spanish versions.

The study was conducted according to the ethical laws of the University of Granada and the World Medical Association and the Declaration of Helsinki (World Medical Association, 2013). Before the beginning of the participation process, people who agreed to take part in the research were informed of the confidentially and the anonymity of the process and the data collected (Lloret-Segura et al., 2014).

The final sample was composed of 241 endurance amateur athletes (38.60% women and 61.40% men), aged 17–61 (*M* = 31.80; *SD* = 9.87). Of these, 29.46% engaged in running and 70.54% engaged in other endurance modalities (e.g., BMX, triathlon, and CrossFit). Regardless of age, participants indicated that they spent 4.13 days per week on training (*SD* = 1.23), spending mainly 60–90 min per session. The participants claimed an experience in the range of 5–10 years in the practice of endurance sports (*M* = 4.86; *SD* = 7.23).

### Measures

The Spanish version of the EAI was used (Terry et al., 2004; Sicilia-Camacho et al., 2013). This instrument was developed according to the “components” of the addiction model, creating one item (e.g., “*Exercise is the most important thing in my life*”) for each one of them (*salience, mood modification, tolerance, withdrawal symptoms, personal conflict*, and *relapse*). The items use a Likert scale ranging from 1 (*Completely disagree*) to 5 (*Completely agree*), where the minimum score is 6 points (*no risk of EA*) and the maximum score is 30 points (*high risk of EA*). According to these scores, the scale allowed us to divide the sample in three categories: asymptomatic (i.e., scores between 0 and 12), symptomatic (i.e., scores between 13 and 23), and at risk of EA (i.e., scores of 24 or more). The internal reliability indicator showed an Alpha Cronbach of 0.90.

The Spanish version of Grit-S was also used (Duckworth and Quinn, 2009; Arco-Tirado et al., 2018). It is an instrument composed by eight items and two factors: Consistency of Interest (e.g., “*New ideas and projects sometimes distract me from previous ones*”) and Perseverance in the Effort (e.g., “*Setbacks don’t discourage me*”). Responses were collected on a 5-point Likert scale ranging from the defined values “*not like me at all*” (1) to “*very much like me*” (5). The final score was obtained by taking the average of the items, where higher scores corresponded with a higher level of grit. The Alpha Cronbach score showed a reliability of 0.84.

The Dark Triad personality traits were measured with the Spanish version of the SD3 (Jones and Paulhus, 2014), adapted for Pineda et al. (2018). This is an inventory composed of 27 items, divided into three first-order factors with the motivation of measuring individual levels of Narcissism (e.g., “*I know that I am special because everyone keeps telling me so*”), Machiavellianism (e.g., “*There are things you should hide from other people because they don’t need to know*”), and Psychopathy (e.g., “*People who mess with me always regret it*”). Furthermore, the scale measure structure described a second-order factor (Dark Triad Personality). Responses were registered using a 5-point Likert scale from 1 (*Strongly disagree*) to 5 (*Strongly agree*). The internal reliability indicators showed an Alpha Cronbach of 0.87 for the second order factor and 0.76, 0.91, and 0.84 for the first order factors, respectively.

### Data Analysis

The internal consistency of the instruments and sample normality and reliability tests were checked through use of the Cronbach alpha and Kolmogorov–Smirnov (K–S), respectively, assuming the suitability of the parametric tests. A descriptive analysis showed the frequencies and measures of central tendency (mean, standard deviation) of the sample distribution. Multivariant analyses (MANOVA over gender and addiction exercise levels) were used to create a differential analysis. A multiple linear and hierarchical regression analysis of EA (Vdependent) (controlled by gender) and a correlation analysis (Pearson) for establishing trends and the relationship between variables were carried out. The data analysis was conducted using the IBM SPSS statistical package version 23.0.

## Results

Table 1 shows the analysis of the variance with the objective of identifying the existence of statistically significant differences in Dark Personality Traits and Grit according to the addiction exercise levels of participants. The K–S test determined that variables were adjusted to the normality assumption. Significant differences were observed for the group of athletes with lower levels of EA (<0.05), specifically for those who showed lower scores with respect to the Grit variable.

**TABLE 1:** Descriptive and differential scores, according gender, and addiction levels.

		Addiction levels								
	K–S	A (*n* = 40)		S (*n* = 162)		RA (*n* = 39)				
		Male (*n* = 22)	Female (*n* = 18)	Male (*n* = 121)	Female (*n* = 41)	Male (*n* = 22)	Female (*n* = 17)	Gender	Addiction level	Gender × addiction level
		
		*M*(*SD*)	*M*(*SD*)	*M*(*SD*)	*M*(*SD*)	*M*(*SD*)	*M*(*SD*)	*F*(*p*)	*F*(*p*)	*F*(*p*)
Perseverance in effort	0.22	3.13 (0.45)	3.04(0.50)	2.98(0.40)	2.93(0.40)	3.23(0.38)	3.25(0.39)	2.32	3.91	4.12^∗^
Consistency in interest	0.23	2.91(0.48)	2.37(0.35)	2.84(0.64)	2.77(0.40)	3.41(0.36)	3.12(0.27)	3.72	4.01^∗∗^	3.14
Narcissism	0.20	2.80(0.45)	3.22(0.95)	2.91(0.35)	2.93(0.38)	3.05(0.45)	2.87(0.35)	3.05^∗^	3.48^∗^	4.03^∗∗^
Machiavellianism	0.20	2.95(0.75)	2.78(1.22)	2.97(0.52)	2.81(0.57)	3.26(0.72)	3.47(0.68)	2.07	2.46^∗^	2.64
Psychopathy	0.18	2.55(0.79)	1.85(0.19)	2.72(0.42)	2.37(0.41)	2.63(0.68)	2.55(0.42)	3.04^∗^	2.06	3.12^∗^

*^∗^*p* < 0.05. ^∗∗^*p* < 0.01. A: asymptomatic; S: symptomatic; RA: risk addiction.*

Differences were also observed between men and women when addiction levels were low (<0.05) in both narcissism (in favor women) and psychopathy (in favor men). However, the comparison scores of the three groups of EA indicated significant differences in Machiavellianism, narcissism, and consistency of interest according to levels of addiction (in favor of the level of risk of addiction), while psychopathy and narcissism were shown to be significant according to gender (in favor of men). Furthermore, significant differences were found between the levels of gender addiction interaction for perseverance in effort (<0.05), narcissism (<0.01), and psychopathy (<0.05).

Addiction levels (*F* = 19.16; *p* < 0.01, η^2^ = 0.03) showed significance in the same way as Gender (*F* = 23.04; *p* < 0.02, η^2^ = 0.06). In detail, the M of Box test was not significant (*F*(_235_,_5__)_ = 4.03; *p* > 0.24). There was no multivariate heteroscedasticity, nor univariate, according to the Levene test [Grit: *F*(_235_,_5__)_ = 1.98; *p* > 0.27; DTP: *F*(_235_,_5__)_ = 1.38; *p* > 0.63; Narcissism: *F*(_235_,_5__)_ = 2.58; *p* > 0.35; Machiavellianism: *F*(_235_,_5__)_ = 1.16, *p* > 0.72; and Psychopathy: *F*(_235_,_5__)_ = 2.53; *p* > 0.35], and this thus assumed compliance with the normal distribution for this type of analysis.

**FIGURE 1:**
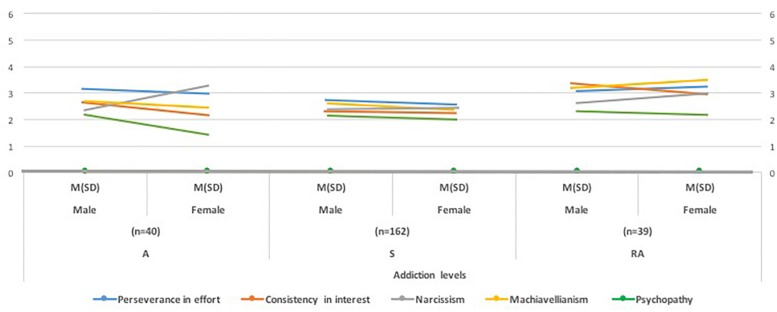
Profiles according gender and addiction levels in endurance sports.

The correlation analyses (Table 2) showed, with low and moderate significance values, that, as age increased, perseverance in effort also increased, and consistency in interest, narcissism, and Machiavellianism in endurance athletes decreased. Perseverance in effort showed a significant inverse relationship to psychopathy, and a positive relationship with consistency in interest and EA. Both Machiavellianism and narcissism showed a direct, moderate, and significant relationship with respect to EA. The number of weekly training sessions showed significant and moderate correlations, both with Dark Features and with the dimensions of Grit and EA (Figure 1).

**TABLE 2:** Correlational analysis (Pearson) between variables.

	*M* (*SD*)	Range	1	2	3	4	5	6	7	8
Age	31.80 (9.8)	(17–65)	–	−0.04	0.20^∗∗^	−0.31^∗∗^	–0.29^∗∗^	–0.27^∗∗^	–0.12	–0.08
Training week (days)	4.13 (1.23)	(1–7)		–	0.51^∗^	0.49^∗∗^	0.46^∗∗^	0.40^∗^	0.47^∗∗^	0.56^∗∗^
Perseverance in effort	2.93 (0.43)	(1–5)			–	0.63^∗∗^	0.56	0.32	–0.43^∗∗^	0.38^∗^
Consistency in interest	2.74 (0.36)	(1–5)				−	–0.45^∗∗^	–0.34^∗∗^	–0.55^∗∗^	–0.47^∗∗^
Narcissism	2.98 (0.38)	(1–5)					−	0.70^∗∗^	0.84^∗∗^	0.54^∗∗^
Machiavellianism	3.17 (0.52)	(1–5)						−	0.70^∗∗^	0.45^∗^
Psychopathy	2.36 (0.45)	(1–5)							−	0.04
Exercise addiction	18.48 (3.56)	(8–30)								−

*^∗^*p* < 0.05. ^∗∗^*p* < 0.01.*

The results of the regression analysis procedure (successive steps forward and weighted by gender) (Table 3) indicated a Model 1 in which athletes showed higher levels of addiction as their weekly training time increased (explaining 33% of the total variance), a Model 2 that added greater indicators of Machiavellianism, thus explaining 28% of the total variance, and a Model 3 that also included an increase of Perseverance in effort scores (explaining 24% of variance).

**TABLE 3:** Regression analysis (stepwise) over addiction exercise.

Dependent variable: addiction exercise	Step	Independent	*B*	*t*	*p*
Model 1. *R* = 0.61; *R*^2^ = 0.33; *F*_5,236_ = 23.59; *p* < 0.00	1	Training week	0.49	2.67	0.02^∗^
		Constant		20.64	
Model 2. *R* = 0.59; *R*^2^ = 0.28; *F*_5,236_ = 26.79; *p* < 0.00	2	Training week	0.29	2.67	0.02^∗^
		Machiavellianism	0.31	3.46	0.00^∗∗^
		Constant		18.74	
Model 3. *R* = 0.49; *R*^2^ = 0.24; *F*_5,236_ = 33.62; *p* < 0.00	3	Training week	0.29	2.67	0.02^∗^
		Machiavellianism	0.37	3.46	0.00^∗∗^
		Narcissism	0.34	3.78	0.01^∗^
		Perseverance in effort	0.31	3.06	0.03^∗^
		Constant		26.37	

*^∗^*p* < 0.05. ^∗∗^*p* < 0.01. Independent variables Model 1 (first step): age; training week. Independent variables Model 2 (second step): age; training week, narcissism; Machiavellianism; psychopathy. Independent variables Model 3 (third step): age; training week, narcissism; Machiavellianism; psychopathy, perseverance in effort, consistency in interest. Weighted least squares regression: weighted by gender.*

## Discussion

Exercise addiction is one of the most representative and negative behavioral patterns that athletes of endurance sports are currently facing, with the highest prevalence rates of EA risk being 14.2% (Di Lodovico et al., 2019). Along with the scientific evidence, different research proposals have been contemplated in order to discover how they function and how EA might be described.

The increase in amateur athletes has, in turn, led to an increase in the likelihood of the appearance of cases of sportsmen and women under risk of EA. With the premise that sports practice is primarily oriented toward competition (including amateur sports), we can consider that a constant demand to improve performance can have an impact on the type of behavior patterns that could favor or protect the manifestation of this type of addictive behavior.

Thus, and through an approach based on the study of uncommon personality traits associated with these behaviors (Dark Traits), we have proposed the analysis of the relationship between Grit (perseverance and passion) and DTP (narcissism, Machiavellianism, and psychopathy) and these excessive behavior patterns in the context of sports (endurance sports) with an aim to explore new ways to explain the appearance and regulation of addiction behaviors by athletes.

This evidence has shown how there exists a moderate risk of EA, and this does not differ much when compared with the most current results found by Szabo et al. (2019), whose study showed that 11.5% of the participants were classified to be at risk of EA based on the EAI-R – a similar percentage (9.0%) to when they use the EDS-R (Lindwall and Palmeira, 2011; Costa et al., 2012, 2013). Based on the results of our research, it appears that the prevalence of being at risk of addiction is ranged between 3 and 5% (Sussman et al., 2011; Berczik et al., 2012; Mónok et al., 2012; Szabo et al., 2013a; Griffiths et al., 2015; Grima et al., 2018; Lukács et al., 2019).

According scientific literature, in the addiction process there seems to be three socio-demographic variables that play an important role in defining the profile of the addicted athlete. These are the athlete’s age, gender, and the number of hours spent training (Lichtenstein et al., 2017).

Regarding gender, the most common results have suggested that men show a higher risk of becoming addicted to exercise (Guszkowska and Rudnicki, 2012; Cook et al., 2013; Szabo et al., 2013b; Cunningham et al., 2016; González-Hernández et al., 2019b), similarly to what happened with the data of the present study. The vast majority of the research related this data to the changing trend in male body care. When this search for the “perfect physical condition,” understood as a physical and mental self-perception (McNamara and McCabe, 2012), is taken to the extreme, exercise (specially in endurance sports) begins to favor the flourishing of addictive behaviors to fulfill the narcissistic desires of the sportsmen (mainly in males) (González et al., 2018). In the present study, men not only scored higher for addiction levels but also for narcissism (grandiosity feelings) and psychopathy (coldness) factors, as some of the studies carried out with high competition athletes have also shown (Ueno et al., 2017). Despite this, studies have recently been conducted on the structure of Dark Traits in athletes, where literature does not provide concrete evidence of invariability between men and women (Vaughan et al., 2019).

For the age variable, our results presented an inverse relationship to levels of addiction, as cited in previous studies (Bruno et al., 2014; Lichtenstein et al., 2014; Grima et al., 2018). At the same time, an inverse relationship between age and Grit and DTP scores (especially narcissism and Machiavellianism) was shown in endurance athletes, which could be explained through the nature of the maturation process of human beings. Over the years, sport and competitive learning experiences have offered athletes a greater ability to regulate their self-knowledge, to cope better with experiences under pressure with less of a need for notoriety or impulsivity, and a greater understanding of situations (Bruno et al., 2014), which helps reduce their compulsive nature and increases the way they control their psychological responses (González-Hernández et al., 2019b).

As with the last of the three indicators for EA – the number of hours spent training – the literature indicated that the athletes who trained more had higher EAI scores (Sicilia and González-Cutre, 2011; Szabo et al., 2013b; Latorre et al., 2016; Lukács et al., 2019; González-Hernández et al., 2019b). Besides this, if signs of narcissism and Machiavellianism increase, perseverance efforts grow too, and the likelihood of EA increases considerably, as similar studies have pointed out in the past (Spano, 2001; Lichtenstein et al., 2014; Miller and Mesagno, 2014; Bircher et al., 2017; Cook et al., 2018). Because of this, a narcissistic personality is often described as a pattern of characteristics present in neurotic and obsessive behavior according to the need to improve and excel when evaluated by others (Wallace and Baumeister, 2002) in the search for achieving any objective and performance. For this reason, in the study presented here, they appear together with narcissistic and Machiavellian features (for example, development of cynical or manipulative attitudes to achieve success or improvement, without valuing personal or social harm) (Miller and Mesagno, 2014).

Despite the few studies that have taken these personality concepts into account to understand the process of EA, González-Hernández et al. (2019b) found similar results on Grit (consistency in interest only) and its condition as a control mechanism. Meanwhile DTP, especially Machiavellianism and narcissism, seems to act as a risk factor. Studied in other contexts (Siah et al., 2010; Maddi et al., 2012; Borzikowsky and Bernhardt, 2018), Grit (general factor) has been shown to be a protection factor against addictive behaviors, such as internet and online game addiction, or in substance use disorders. Meanwhile, DTP studies (González et al., 2018) have linked narcissism indicators with EA (Basson and Myers, 2001; Bruno et al., 2014; Lichtenstein et al., 2014). Nevertheless, Machiavellianism has been associated with athletes with greater abilities of persuasion, both toward other people (including manipulation) and toward themselves (Deak et al., 2017; Tomankova, 2017).

## Limitations

It is important to recognize the limitations of this research and to consider future research directions. First, in spite of the number of participants being acceptable, we acknowledge that we could have found stronger results with a larger number of athletes. In line with this limitation, the sample distribution regarding the addiction levels was not balanced, although the tendency shown in our data is very similar to other studies conducted on EA; we think it would be of significant interest to try to achieve a similar number of cases for each level. Secondly, since this study only had one sport that was analyzed, it would be interesting to examine if EA symptoms differ by sport modality. And thirdly, even with attention to detail in the data collection procedure, a possible bias regarding the desirability of pleasing responses associated with DTP could result in a limitation in the interpretation of the data. Although the scale offered statistics for this and for most of the studies in which it had been used, for this particular case we only checked the relationship between EA and Grit. It could be interesting to include other explanatory variables or personality traits such as perfectionism or impulsivity.

## Conclusion

This research aimed to analyze and identify the role of two concepts related to personality – the Dark Triad (narcissism, Machiavellianism, and psychopathy) and Grit (perseverance and passion) – and their relationship with EA in endurance sports. These sport modalities demand not only a great physical dedication (hours of training) but also significant psychological efforts. It is important to know about the beliefs and personality traits of these sportsmen and women and know if they can act as a protector or facilitator of exercise addictive behaviors.

We can effectively see what seems to be a degree of accordance in regards to the way personality plays an important role in the manifestation of addictive exercise behaviors due to the significant differences that were found in Dark Personality Traits and Grit according to the levels of EA in participants. In this sense, higher levels of Grit could be associated with lower levels of addiction. Meanwhile, the opposite relationship occurs when the likelihood to suffer a “risk of addiction” increases with the presence of the Dark Triad, especially with the manifestation of the Machiavellianism trait.

Considering the role of the age, it seems that as runners get older they have higher levels of grit, although the manifestation of Dark Traits and EA is reduced. That fact confirms that when an athlete can manage the direction of their thoughts and guides them toward a conscious practice according to their reality. Grit could act as a factor of balance in self-regulation.

The significant differences found among both sexes, showed higher addiction levels in favor of sportsmen and higher indicators of grit, narcissism, and psychopathy. This proves that sportsmen were more impulsive and thrill-seeking at the same time they display lower levels empathy. Besides this, they showed a more competitive behavior and further dominance and superiority when they practice sports.

Finally, the predictive models confirmed the importance of the time spent training (number of training sessions per week) and the Machiavellianism, regarding the modulation of the manifestation of EA. As we have already indicated, the cognitions and their manipulative character play an important role in the relationship with the levels of EA, up to the point that the athletes feel the imperative need to train more (compulsion) to reduce their anxiety, which, at the same time, acts as a control mechanism (obsession).

## Data Availability Statement

The datasets generated for this study are available on request to the corresponding author.

## Ethics Statement

The studies involving human participants were reviewed and approved by the University of Granada. Written informed consent to participate in this study was provided by the participants’ legal guardian/next of kin.

## Author Contributions

AN: planning, development, data collection, and document writing. JG-H: planning, development, data collection, statistical analysis, and document writing. MT-G: final writing review and conclusions and theoretical frame review.

## Conflict of Interest

The authors declare that the research was conducted in the absence of any commercial or financial relationships that could be construed as a potential conflict of interest.
